# Supplementary material for: Predictors of Development of Hepatorenal Syndrome in Hospitalized Cirrhotic Patients with Acute Kidney Injury
Source: J Clin Med. 2021 Nov 29;10(23):5621. doi: 10.3390/jcm10235621 (PMC8658275; doi:10.3390/jcm10235621)
Supplement: Supplementary file 1 [file jcm-10-05621-s001.zip › jcm-1469596-supplementary.pdf]

**Supplemental Table S1.** Odds ratio for development of HRS-AKI based on ascites grade on admission

| Ascites Grade | Crude OR (CI) | p-Value |
|---|---|---|
| 1 | 3.2 (1.2–8.8) | <0.05 |
| 2 | 13.1 (5.7–30.2) | <0.05 |
| 3 | 25.9 (10.6–63.4) | <0.05 |

**Supplemental Table S2.** Variables associated with the development of HRS in cirrhotic patients admitted with Acute Kidney Injury stratified by the presence of chronic kidney disease.

| Variables | | Development of Hepato-Renal Syndrome | | Crude OR (CI) | p-Value |
|---|---|---|---|---|---|
| | | Yes N (%) | No N (%) | | |
| Cr >2.5 mg/dL in patients with normal kidney function at baseline | Yes | 19 (40%) | 61 (16%) | 4.3 (2.2–8.2) | <0.05 |
| | No | 28 (60%) | 326 (84%) | | |
| Cr>2.5 mg/dL in patients with chronic kidney disease | Yes | 2 (40%) | 33 (37%) | 1.2 (0.2–7.2) | 0.694 |
| | No | 3 (60%) | 57 (63%) | | |
